# Defective proviruses significantly impact viral transcription and immune activation in men and women with HIV-1 subtype C in rural South Africa

**DOI:** 10.3389/fimmu.2024.1484358

**Published:** 2024-11-26

**Authors:** Ninée V. E. J. Buchholtz, Lucas E. Hermans, Chijioke N. Umunnakwe, Marieke M. Nühn, Regina Voss, Emma Need, Neeltje A. Kootstra, Irma Maurer, Dorien C. M. de Jong, Jori Symons, Hugo A. Tempelman, Annemarie M. J. Wensing, Monique Nijhuis

**Affiliations:** ^1^ Translational Virology, Department of Medical Microbiology, University Medical Center Utrecht, Utrecht, Netherlands; ^2^ Ndlovu Laboratories, Ndlovu Research Center, Ndlovu Academic Department, Ndlovu Care Group, Elandsdoorn, South Africa; ^3^ Department of Experimental Immunology, Amsterdam institute for infection and immunity, Amsterdam UMC Location University of Amsterdam, Amsterdam, Netherlands; ^4^ Translational Virology, Department of Global Public Health and Bioethics, Julius Center for Health Sciences and Primary Care, University Medical Center Utrecht, Utrecht, Netherlands; ^5^ Ezintsha, Faculty of Health Sciences, University of the Witwatersrand, Johannesburg, South Africa; ^6^ HIV Pathogenesis Research Unit, Faculty of Health Sciences, University of Witwatersrand, Johannesburg, South Africa

**Keywords:** HIV-1 subtype B and C, IPDA, male and female, reservoir, quantification, HIV-1 transcription, low-and middle-income countries

## Abstract

**Introduction:**

The main obstacle to achieving an HIV-1 cure is the proviral reservoir. To promote equity in HIV cure strategies, it is crucial to study the viral reservoir of the predominant HIV-1 subtype C in both women and men. Therefore, we investigated the dynamics of the (intact) viral reservoir in relation to plasma viral load (VL), CD4^+^ T cell count, and immune activation before and during 96 weeks of successful antiretroviral therapy (ART).

**Methods:**

Eighty-two participants (62% female) newly initiating ART in a rural clinic in South Africa were included in the study. Blood samples were collected at baseline, week 48, and week 96, and CD4 count was determined. Plasma was used for VL and immune marker analyses, while isolated peripheral blood mononuclear cells (PBMCs) were used for the quantification of cellular multiple spliced HIV-1 RNA (msRNA) and the intact proviral DNA assay. For the longitudinal analyses on ART, we selected only those participants who durably suppressed their VL to <200 copies/mL during 48 (n=65) and/or 96 (n=60) weeks of treatment.

**Results:**

At ART initiation, the median CD4 count was 234 cells/mm3 and VL was 68,897 copies/mL. Interestingly, at baseline the number of defective proviruses was significantly correlated with VL (p<0.0001), msRNA (p<0.0001), CD4 count (p=0.0008), CXCL10 (p=0.0003) and TNF-α (p=0.0394). During successful ART, a significant decrease of both the intact and defective proviral reservoir was observed (p<0.0001). The decrease of the intact proviral reservoir was more profound compared to the defective fraction after 96 weeks of therapy. In addition, a significant decrease in cellular msRNA and IL-6, IL-7, TNF-α, sCD14, sCD163, CCL2, CXCL10, and CRP was detected.

**Discussion:**

This study underscores the significant relationship observed prior to therapy initiation between the number of defective proviruses, viral transcription/production and their association with immune response indicators such as CD4 count, CXCL10, and TNF-α. Furthermore, the observation of a less pronounced decrease of the defective proviral DNA highlights the importance of addressing both intact and defective proviruses in therapeutic strategies to enhance clinical outcomes for people with HIV-1. Together, these findings suggest a significant role of the defective proviruses in HIV-related disease progression.

## 1 Introduction

Since the discovery of HIV-1 over four decades ago, extensive efforts have been dedicated to unraveling its complex biology and developing effective treatments. Significant advancements in antiretroviral therapy (ART) have transformed HIV-1 infection from a fatal diagnosis to a manageable chronic condition. Despite extensive research exploring multiple avenues towards a cure, only rare instances of stem cell transplantation with a CCR5Δ32 mutation have led to viral eradication, but a definitive cure on a global scale remains elusive (1–5). Viral diversity, the generation and persistence of viral reservoirs accompanied by the challenges in targeting these latent HIV-1 reservoirs have posed formidable obstacles herein (6–9), and it remains a highly complex topic within the field of HIV-1 research.

Although HIV-1 subtype C is the most prevalent subtype worldwide with ~47% of all HIV-1 infections, most research has focused on HIV-1 subtype B which constitutes only ~12% of all global infections (10–12). Subtype B predominantly occurs in Western and Central Europe, North and Latin America, and the Caribbean, whereas subtype C is primarily found in Eastern and Southern Africa (13). Moreover, subtype B exhibits a higher prevalence among men compared to women, while subtype C shows the opposite pattern (13, 14). The distinct genetic variations between subtypes are known to influence, in some cases, viral transmission, efficacy of ART, and disease progression. In a multicenter, prospective observational study conducted in Switzerland involving 1,057 people with HIV (PWH) on ART from diverse demographics and ethnicities, it was demonstrated that individuals infected with HIV-1 subtype C exhibited lower total HIV-1 DNA levels. They also showed a faster decay of the reservoir compared to those infected with HIV-1 subtype B (15). Despite this, there remains a lack of data on different viral subtypes, and research on viral reservoir dynamics has been notably understudied in women, even though women account for 60% of new infections in sub-Saharan Africa (16). The available data on biological sex differences are limited and contradictory in findings regarding reservoir size. While some studies indicate no differences between sexes, others suggest that women may have a lower frequency of inducible virus (17–19), underscoring the necessity to expand our understanding of the reservoir beyond men infected with subtype B.

HIV-1 establishes viral reservoirs early after infection, most likely already within the first few days or weeks after transmission (20). The virus enters a state of dormancy within infected (immune) cells where it remains largely invisible to the immune system (21, 22). These reservoirs consist not only of intact and potentially replication-competent proviruses but also of proviruses with deletions and hypermutations that are considered defective and therefore unable to replicate. The current generation of antiretroviral compounds can inhibit viral replication but unfortunately are unable to inhibit viral transcription, protein synthesis, and virus production from infected cells. Currently, most HIV-1 cure research focuses on eliminating the intact reservoir as it can induce new rounds of infection in the absence of ART. However, this reservoir encompasses only about 3% of the total reservoir in individuals with HIV-1 subtype B infections on suppressive long-term ART (23). In contrast, the defective fraction encompasses by far the larger portion of the total proviral DNA load (9, 24). In untreated individuals, these proportions are more variable, with recent studies indicating intact fractions ranging from ∼40% to ∼65% (25, 26).

Several methodologies are available for evaluating the proviral reservoir. One of the recent advancements is the intact proviral DNA assay (IPDA), a high-throughput assay that distinguishes between defective and intact provirus, thereby enabling precise quantification of the potentially replication-competent reservoir (23). This assay utilizes two fluorescent signals, one at the 5’ packaging region (*psi*) and another at the 3’ envelope region (*env*) (23). We recently developed an adapted IPDA to encompass both HIV-1 subtypes B and C (27). While the IPDA provides insights into the size of the (intact) proviral reservoir, viral RNA transcription serves as a valuable marker of its activity. RNA products such as unspliced, single-spliced, and multiple-spliced (ms) RNA are indicative of viral transcription, with msRNA particularly predictive of virus production (28). Together, the IPDA and msRNA assays offer a comprehensive characterization of the proviral reservoir dynamics.

To address the gap in HIV-1 cure research, our study aimed to investigate the dynamics and activity of both defective and intact reservoirs during ART in a well-characterized cohort of rural South African women and men living with HIV-1 subtype C. Specifically, we explored the relationships between the intact and defective proviral DNA and variables such as viral load (VL), CD4^+^ T cell counts, msRNA, and plasma immune markers among study participants. Additionally, we analyzed how these parameters evolved over the course of 96 weeks of successful ART.

## 2 Methods

### 2.1 Study population

A total of 501 trial participants aged 18 years and older were included in the ITREMA randomized clinical trial (ClinicalTrials.gov Identifier: NCT03357588). This study was an open-label randomized evaluation of different strategies for treatment monitoring, during which both study arms received standard-of-care first-line antiretroviral treatment and were followed up for a period of 96 weeks (29). ART consisted of either Tenofovir Disoproxil Fumarate (TDF), Emtricitabine (FTC), and Efavirenz (EFV); or Abacavir (ABC), Lamivudine (3TC), and EFV. The study ran between June 2015 and January 2019 at Ndlovu Medical Centre in Elandsdoorn (Limpopo province, South Africa), and received ethical approval from the University of Pretoria Human Research Ethics Committee (Ref Number: 69/2015) and the Limpopo Department of Health (Ref No 4/2/2).

A total of 207 participants in the ITREMA trial reported to be therapy naïve and were initiating ART at trial enrolment. We aimed to collect PBMCs of 100 of these participants. For 87 participants, PBMCs were successfully isolated and stored. Subsequently, HIV-RNA load testing was performed on the enrolment plasma sample of all participants, and for those with a VL<1,000 copies/mL at enrolment. An enrolment plasma sample was tested for the presence of undisclosed use of antiretroviral drugs EFV and lopinavir. These were the prevailing available anchor drugs in South Africa at the time of the trial. Four participants were excluded because their EFV or lopinavir levels exceeded the limit of detection (of 0.05 mg/L and 0.06 mg/L respectively) (29). In addition, one participant was retrospectively excluded as PBMC collection had occurred several days after ART initiation. This resulted in 82 samples being available for analysis. For analyses of viral reservoir dynamics over time on ART, we selected only those participants who had a durable suppression of their viral load <200 copies/mL during 48 (n=65) and/or 96 (n=60) weeks of follow-up on ART.

### 2.2 Collection of data samples

Samples were taken from all participants at baseline, 48 weeks, and 96 weeks after therapy initiation. After blood draw, immediate separation of plasma and isolation of PBMCs was performed using Ficoll-paque™ and a Leucosep® tube. Plasma was immediately stored at -80°C onsite. PBMCs were pelleted by centrifugation and counted using the Beckman Coulter Cell counter. Isolated cells were immediately stored in a mixture of Fetal Bovine Serum, Iscove’s Modified Dulbecco’s Medium and dimethyl Sulfoxide at -135°C onsite.

### 2.3 Viral load and drug exposure testing

Quantitative measurement of HIV-1 RNA was performed on plasma collected in plasma preparation tube vacutainers using the Roche COBAS^®^ AmpliPrep/COBAS^®^ TaqMan^®^ HIV-1 Test, version 2.0 (Roche Molecular Systems, USA). Results above 50 copies/mL were reported quantitatively, and results below this threshold were reported as “lower than detectable limit”. Viral load measurement was performed in real-time during trial visit timepoints, and retrospectively on stored ethylenediaminetetraacetic acid-derived samples of all participants at ART initiation.

Drug exposure testing was performed retrospectively on samples collected at the timepoint of ART initiation in patients who had either a VL <1000 copies/mL at ART initiation or had reported prior exposure to ART. Drug exposure testing was performed for EFV and lopinavir, which were the two base drugs that were in use locally according to contemporary local ART guidelines. Testing was performed on ethylenediaminetetraacetic acid-derived plasma using a validated immunoassay that was implemented at Ndlovu Medical Centre as described previously (29).

### 2.4 Intact/defective HIV-1 DNA quantification

Genomic DNA was isolated from PBMC samples at baseline, 48 and 96 weeks of therapy using the DNeasy Blood and Tissue Kit (Qiagen), according to manufacturer’s guidelines. HIV-1 IPDA was developed as a multiplex digital droplet (dd)PCR assay (23). We optimized the IPDA to correctly quantify HIV-1 subtype B and C (**Supplementary Table 1**) (27). In each ddPCR reaction, primers, probes, the restriction enzyme XhoI (20 U/µl, NEB), and the ddPCR Supermix for probes (No dUTP) (Bio-Rad) were mixed with the DNA according to manufacturer’s protocol (Bio-Rad). Samples with less than 10,000 droplets were excluded from further analysis. As a positive control, JLat 15.4 cells (NIH AIDS Reagent Program, 9848) were used (30). DNA isolated from PBMCs of HIV-negative donors was used as a DNA control and water as a no-template control. Cycle conditions were performed as described in the original IPDA (27). Results were analyzed using Quantasoft version 1.7.4. The threshold was manually set based on positive and negative control samples and equally applied throughout all experiments. Samples were included if the DNA shearing index was <0.50, and if they met the cutoff of at least 100,000 cells tested or if at least 7 copies were detected in each single fluorescent signal, or 6 copies in the double fluorescent signal (27). If no copies were detected but the cutoff value of at least 100,000 cells was met, a detection limit of 2.86 for single, and 1.96 for double positive signals, was used.

### 2.5 msRNA quantification

Total RNA was isolated from PBMC samples at baseline, 48, and 96 weeks after therapy using the RNeasy Mini Kit (Qiagen), according to manufacturer’s guidelines. cDNA was generated using a gene-specific reverse primer (**Supplementary Table 1**) and the TaqMan Reverse Transcription Reagents (Invitrogen, N8080234). HIV-1 RNA was quantified using the ddPCR targeting the HIV-1 subtype C msRNA region (**Supplementary Table 1**). The ddPCR was performed according to the manufacturer’s protocol (Bio-Rad). Samples with less than 10,000 droplets were excluded from further analysis. As a positive control, gblocks designed for msRNA subtype C were used. As no-template controls, water and RNA of HIV-negative donors converted to cDNA were used. Cycling conditions were used in line with the manufacturer’s protocol except for the annealing temperature, which was adjusted from 60°C to 58°C. msRNA copies were quantified based on RNA input per sample. If no copies were measured, a limit of detection of 1 copy/µg RNA was used. Analysis of the results was performed using Quantasoft version 1.7.4.

### 2.6 Plasma biomarker analysis

Luminex was used to determine the plasma levels of CRP, sCD14, CCL2, sCD163, CXCL10, IL-2, IL-6, IL-7, IL-12, and TNF-α (Bio-Techne, Minneapolis, MN, USA; LXSAHM-08 and LXSAHM-02). Luminex was performed according to the manufacturer’s instructions.

### 2.7 Data analysis and statistics

Cross-sectional correlations between continuous parameters were performed using linear regression. For comparisons between sexes, the Unpaired t-test or Mann-Whitney U test was used. For assessment of reservoir dynamics and activity over time during ART, linear mixed effects models were used. For each model the parameter of interest was set as the outcome variable, and covariables of interest (biological sex, baseline VL, baseline msRNA, baseline CD4^+^ T cell count and baseline total proviral DNA) were entered in addition to a time variable as fixed-effect covariables, with the participant ID entered as a random effect. The full linear mixed effects analysis as performed in R is presented in **Supplementary Table 2**. All variables, except CD4^+^ T cell counts, were log-transformed. Statistical analyses were performed using GraphPad Prism v10.2.1 (GraphPad Software, San Diego, California, USA) and R 4.1.2. Statistical tests are indicated within the figure legends, p-values of < 0.05 were considered significant. Tables were composed using Excel (Microsoft Office), and graphs with GraphPad Prism v10.2.1 (GraphPad Software, San Diego, California, USA).

## 3 Results

The study population comprised 82 individuals with a median age of 39 years, with 62% being female (n=51) initiating antiretroviral therapy (**Table 1**). All participants were living with HIV-1 subtype C. Initial ART consisted of TDF, FTC, and EFV for 99% (81/82) of participants and ABC, 3TC, and EFV for 1% (1/82) of participants. The median CD4^+^ T cell count at ART initiation was 234 cells/mm^3^ (interquartile range (IQR): 73; 371), of which 34 participants presented to the clinic with a CD4^+^ T cell count <200 cells/mm^3^. The median VL was 68,897 copies/mL (IQR: 20,031; 166,481).

**Table 1:** Baseline characteristics.

Baseline characteristics	n=82
Age, median in years (IQR)	39 (31; 47)
Sex, female; male	51; 31
HIV-1 subtype	C
ART regimen	TDF/FTC/EFC (n=81); ABC/3TC/EFV (n=1)
CD4^+^ T cell count, median in cells/mm^3^ (IQR) Log^10^ cells/mm^3^	234 (73; 371) 2.22
Plasma viral load, median in copies/ml (IQR) Log^10^ copies/mL	68,897 (20,031; 166,481) 4.56

### 3.1 Baseline reservoir characterization

**Figure 1:**
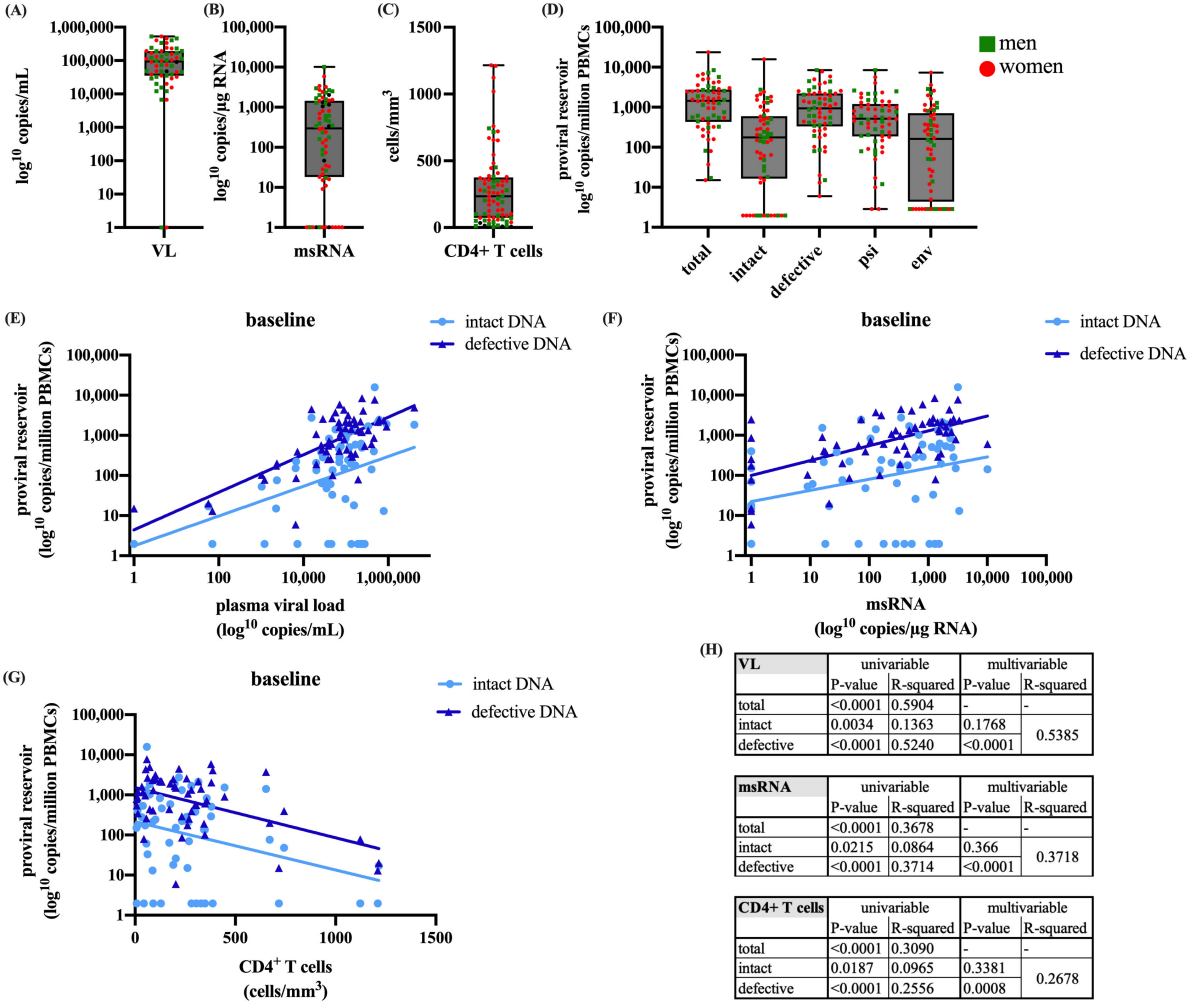
Presents the baseline characteristics of **(A)** plasma viral load, **(B)** cell-associated msRNA, **(C)** CD4^+^ T cells, and **(D)** IPDA quantifications of total, intact, defective, psi, and env DNA. Additionally, linear regressions are presented of intact and defective proviral DNA at baseline with **(E)** plasma viral load, **(F)** cell-associated msRNA, and **(G)** CD4^+^ T cells. Table **(H)** shows the univariable and multivariable p-values and the R^2^. The boxplots present (from top to bottom) the maximum, third quartile, median, first quartile, and minimum values. For the linear regression model Values of p<0.05 were considered significant. The dataset was log transformed and the analysis was performed with a simple linear regression in R and GraphPad.

We examined the relation of intact versus defective proviruses with RNA production and disease progression at baseline. The baseline distributions of the VL, msRNA, CD4+ T cells, and proviral DNA are shown in **Figures 1A–D**. A significant correlation was observed between the size of the total proviral DNA reservoir and VL at baseline (p<0.0001, data not shown). Intriguingly, the correlation with VL at baseline was stronger for defective than for intact proviral DNA (p<0.0001 vs. p=0.0034) with the R^2^ value indicating a better model fit for defective proviral DNA (R^2^ = 0.5240 vs. R^2^ = 0.1363) (**Figure 1E**). Furthermore, reservoir activity measured by cell-associated msRNA levels demonstrated a significant correlation with total proviral reservoir size (p<0.0001, data not shown). Once again, this correlation was more pronounced for defective compared to intact proviral DNA (p<0.0001, R^2^ = 0.3714 vs. R^2^ = 0.0864, p=0.0215) (**Figure 1F**). We also observed a significant inverse correlation between baseline total proviral reservoir size and CD4^+^ T cell count (p<0.0001, data not shown). Consistent with the other observations, this correlation was stronger for defective proviral DNA compared to intact proviral DNA (p<0.0001, R^2^ = 0.2556 vs. p=0.0187, R^2^ = 0.0965) (**Figure 1G**). These findings underscore the significant contribution of the defective proviral reservoir to HIV-1 RNA production and its inverse relationship with CD4^+^ T cell count.

For VL, msRNA, and CD4^+^ T cell count, the univariable analysis demonstrates a significant correlation with both intact and defective proviral DNA, with a higher R^2^ for defective proviral DNA. To determine which variable is a better predictor, we performed a multivariable analysis. The results indicate that the significance with intact proviral DNA is lost, while it is retained with defective proviral DNA (**Figure 1H**). This suggests that the correlations with defective proviral DNA represent a more independent effect, not masked by other factors, making it a better predictor and more prominent in influencing the outcomes measured by VL, msRNA, and CD4^+^ T cell count.

Moreover, we examined the impact of CD4^+^ T cell count before the start of ART on VL, msRNA, intact proviral DNA and defective proviral DNA. When participants were divided into two groups based on CD4^+^ T cell counts (<200 and >200 cells/mm^3^), those with counts below 200 showed significantly higher viral loads, msRNA levels, and defective DNA (**Supplementary Figure 1**). However, no significant difference in intact proviral DNA was observed between the two groups (**Supplementary Figures 1A–D**).

### 3.2 Proviral reservoir over time

**Figure 2:**
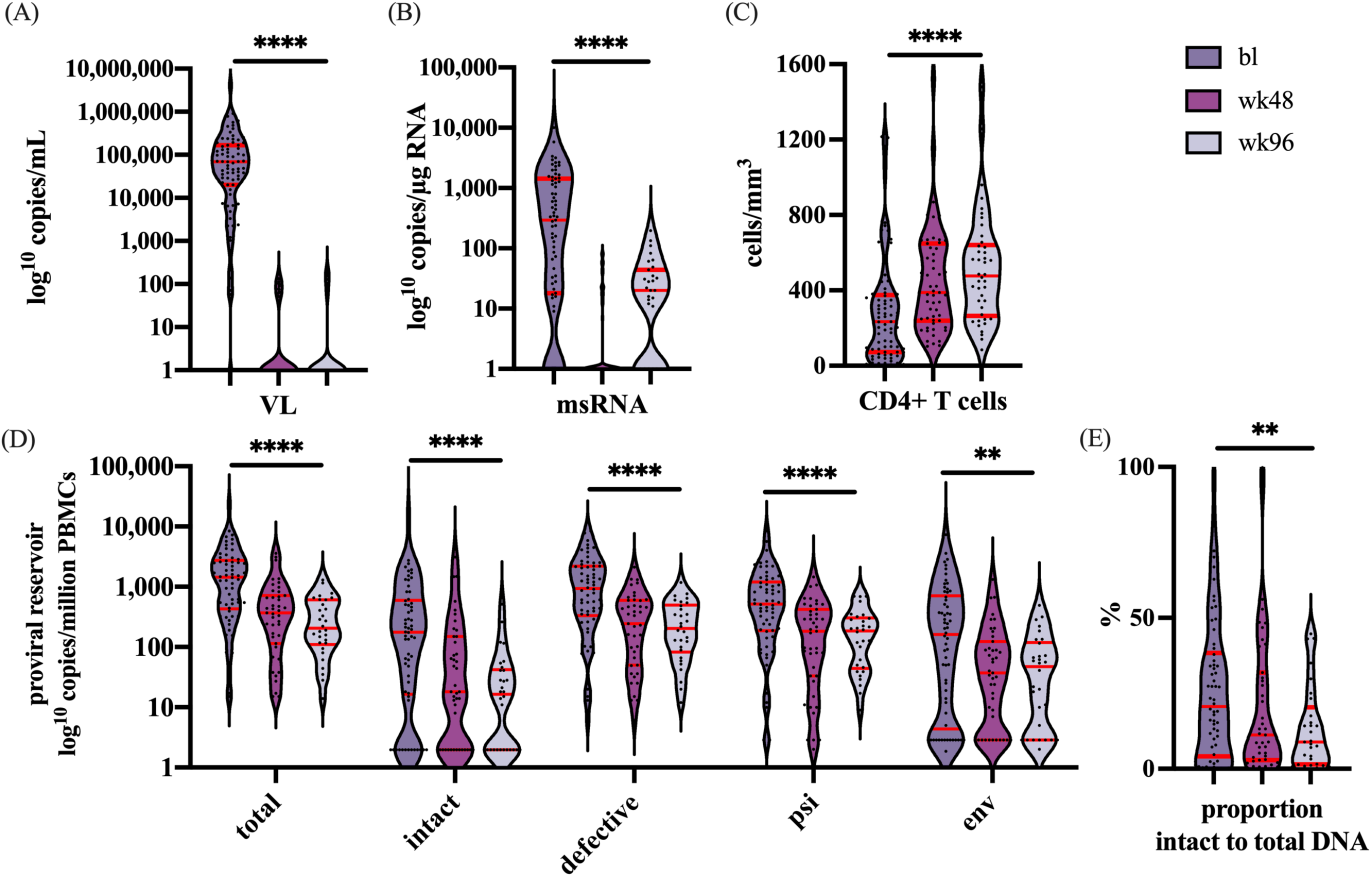
Represents the dynamics over time for the following parameters: **(A)** The VL in copies/mL, **(B)** msRNA in copies/µg RNA, **(C)** CD4^+^ T cells in cells/mm^3^, **(D)** the number of copies/million PBMCs of total, intact and defective proviruses, and the proviruses with only the psi or env region. **(E)** The percentage decline of the intact proviral reservoir related to the total proviral reservoir. Significance is indicated as: **p<0.001, ****p<0.0001. The violin plot depicts the distribution of the data showing the individual values. The red lines indicate (from top to bottom) the third quartile, the median, and the first quartile. Graphs were designed in GraphPad, and the analysis performed with a linear mixed-effects model in R. For every outcome measured over the time a correction was performed with the remainder co-variables at baseline: Sex, CD4^+^ T cells, VL, msRNA, and total proviral DNA.

To evaluate the impact of ART on the size and activity of the viral reservoir in PBMCs, we assessed plasma RNA, cell-associated msRNA, CD4^+^ T cell count, and HIV-DNA levels at baseline (n=82), 48 weeks (n=65), and 96 weeks (n=60) of treatment in patients who achieved durable suppression (<200 copies/mL) during treatment (**Figure 2A**). In these participants, we observed a significant decrease in cell-associated msRNA (**Figure 2B**) and a significant increase in the number of CD4^+^ T cells (**Figure 2C**) (both p<0.0001).

Furthermore, during the first year of treatment, a significant reduction was observed for total, defective, and intact proviral DNA (**Figure 2D**). Interestingly, after one year of ART the intact proviral DNA continued to decline whereas no further decline was observed for the defective proviral DNA. When analyzed separately, significant reductions in both the intact and defective fraction were observed (p<0.0001 for both). However, when the fraction of intact provirus to total proviral DNA was analyzed, this fraction was noted to decline over time (p=0.0059), indicating that the decay of intact proviral DNA was more rapid than that of defective proviral DNA (**Figure 2E**). These results indicate a more pronounced decrease of the intact reservoir compared to defective proviruses.

### 3.3 Immune activation

HIV-1 infection is characterized by its ability to target and compromise the immune system, progressively weakening it. Consequently, we aimed to examine the relationship between the size and dynamics of the viral reservoir and immune activity.

**Figure 3:**
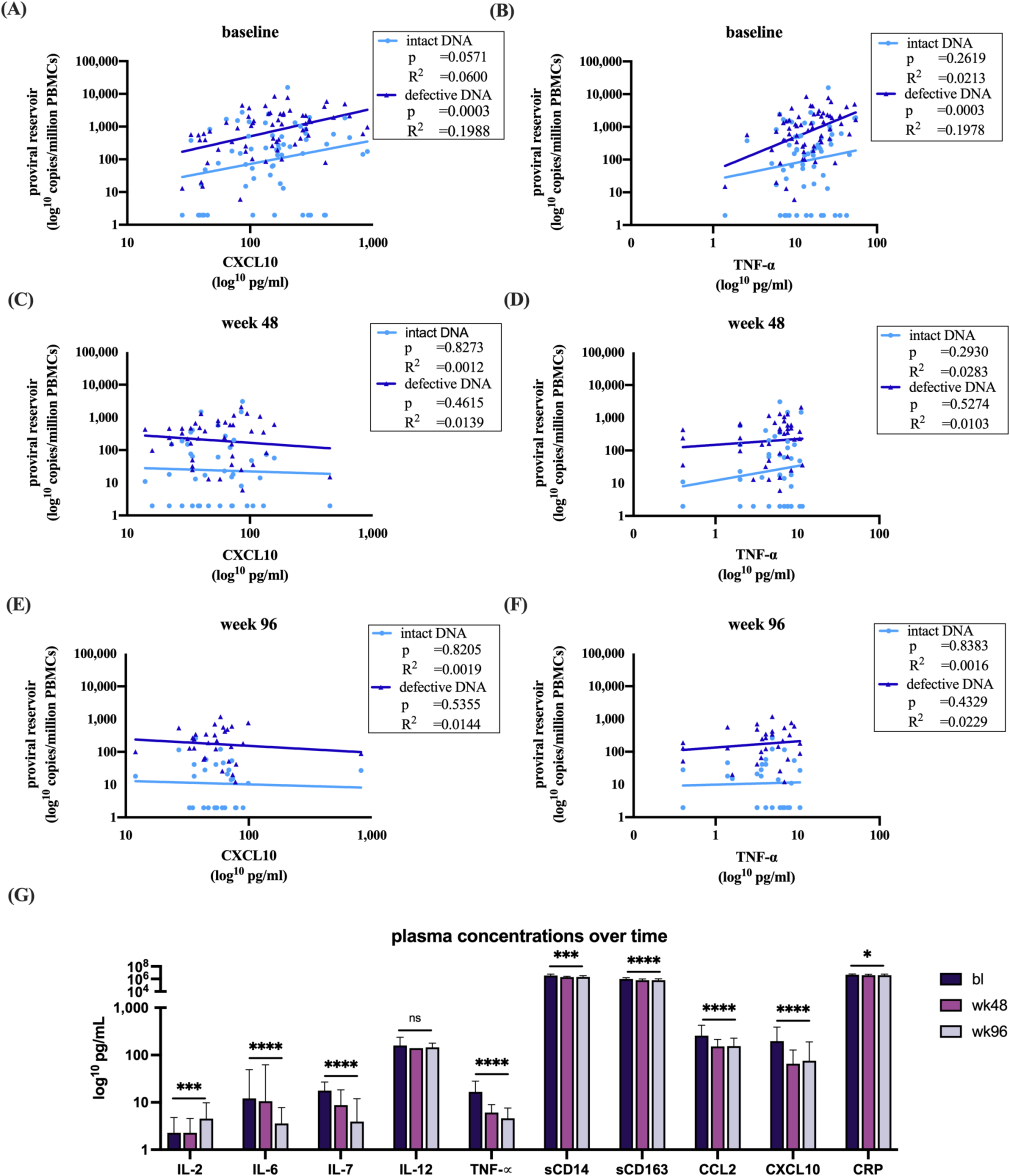
Analysis of plasma sample with intact and defective proviral DNA and **(A)** at baseline correlations with CXCL10 **(B)** baseline correlations with TNF-α **(C)** week 48 correlations with CXCL10 **(D)** week 48 correlations with TNF-α **(E)** week 96 correlations with CXCL10 **(F)** week 96 correlations with TNF-α. **(G)** Dynamics of the measured immune markers at bl, 48 and 96 weeks after start of therapy. All datasets were log transformed. Analysis overtime was performed with a linear mixed effects model in R. Correlations were analyzed with a linear regression model in GraphPad. Figures were made in GraphPad. *p<0.05, ***p<0.001, ****p<0.0001. The bars represent mean ±SD. Abbreviations: Interleukin (IL), Tumor Necrosis Factor alpha (TNF-α), cluster of differentiation (CD), chemokines C-C motif chemokine ligand 2 (CCL2), C-X-C motif chemokine 10 (CXCL10), c-reactive protein (CRP).

Within this cohort, significant baseline correlations were observed between the size of the total viral reservoir and two cytokines (CXCL10; TNF-α) (p=0.0002, data not shown). The chemokine CXCL10, shows a significant correlation with the defective proviruses, whereas no significant correlation was found with the intact reservoir (p=0.0003, R^2^= 0.1988 vs. p=0.2418, R^2^= 0.0279) (**Figure 3A**). TNF-α, a proinflammatory cytokine responsible for immune regulation and apoptosis, showed a similar pattern, significantly correlating with the defective proviruses but not with the intact reservoir (p=0.0394, R^2^= 0.0838 vs. p=0.0571, R^2^= 0.0600) (**Figure 3B**) (31). These findings suggest a link between the defective proviruses and immune activation in the absence of treatment. However, no correlations were found between proviral DNA and the cytokines at later timepoints during effective antiretroviral therapy (**Figures 3C–F**).

Additionally, we investigated the impact of baseline CD4^+^ T cell counts below and above 200 cells/mm³ on CXCL10 and TNF-α levels prior to the initiation of ART. Both immune markers were found to be significantly elevated in the group with baseline CD4^+^ T cell counts below 200 cells/mm³ (**Supplementary Figures 1E, F**).

Furthermore, a clear effect of suppressive ART on immune activation was demonstrated by decreased plasma immune markers. Notably, IL-6, IL-7, TNF-α, sCD14, sCD163, CCL2, CXCL10, and CRP all exhibited significant reductions following therapy initiation, except for IL-12 where no difference was observed (**Figure 3G**). In contrast to the other immune markers, IL-2, which promotes the development of T regulatory cells, showed a significant increase (**Figure 3G**) (32).

### 3.4 Biological sex differences

**Figure 4:**
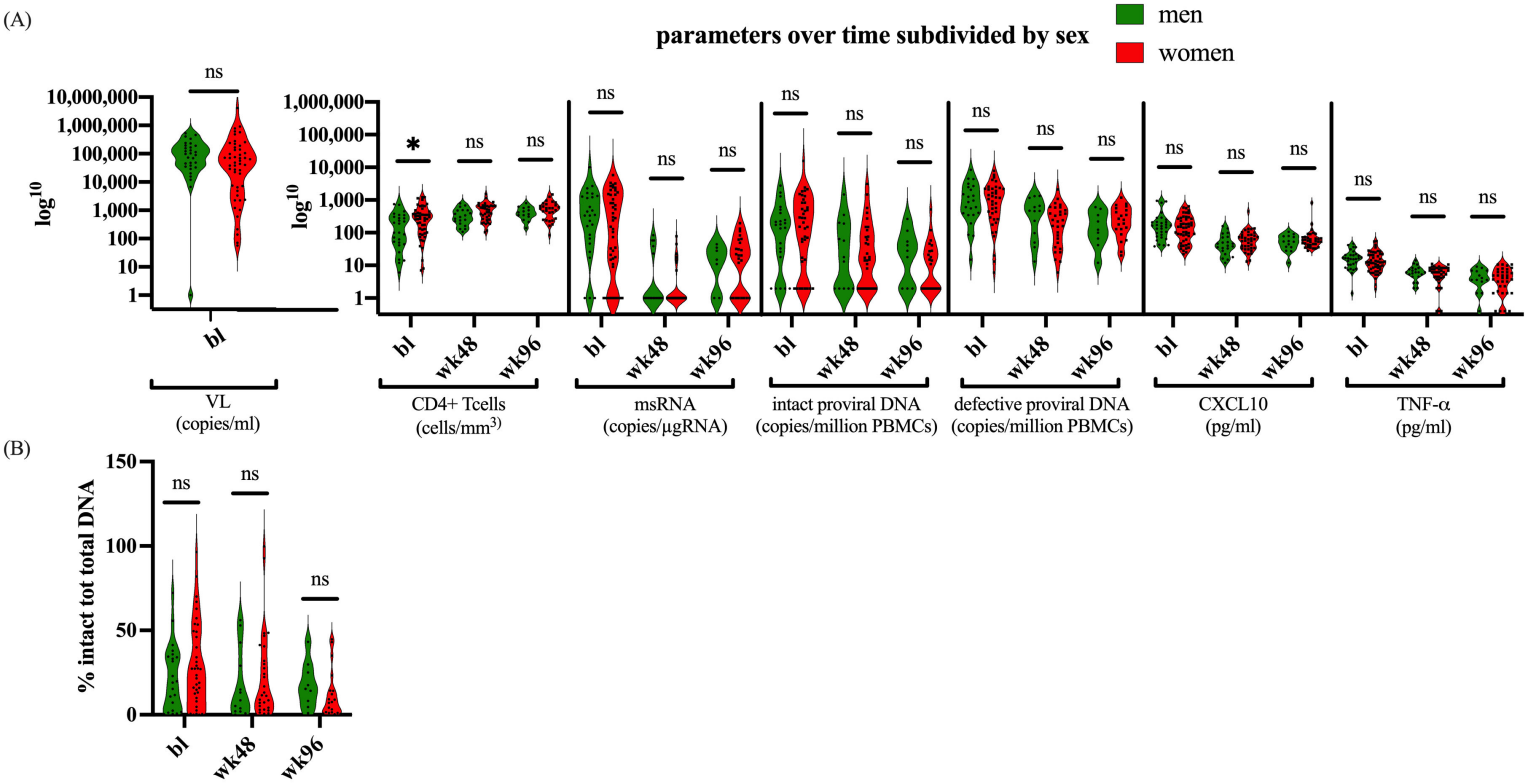
Parameters divided by biological sex. **(A)** Depicts the parameters CD4^+^ T cells, msRNA, total proviral DNA, intact proviral DNA, CXCL10, and TNF-α, at bl, 48 weeks, and 96 weeks after therapy for males and females. **(B)** shows the proportion of intact proviral DNA in relation to total proviral DNA between males and females. The dataset was log transformed, and the analysis was performed with an Unpaired t test or a Mann-Whitney U test in GraphPad. Values of p<0.05 were considered significant. The violin plot depicts the distribution of the data showing the individual values.

We subsequently set out to explore the dynamics of HIV-1 and the proviral reservoir within our participant group, focusing on potential biological sex differences. Notably, women comprised 62% of the participants at baseline, 66% at week 48, and 68% at week 96. Upon comparing CD4^+^ T cell counts between women and men, we observed that at baseline, males had significantly lower CD4^+^ T cell counts than females (p=0.0212) (**Figure 4A**).

However, no significant differences in CD4^+^ T cell count were found at later timepoints. Moreover, our cohort showed no differences in VL, msRNA levels, proviral DNA, or the cytokines CXCL10 and TNF-α (**Figure 4A**). There were also no differences between males and females in the ratio of their intact to total proviral DNA (**Figure 4B**). Furthermore, biological sex did not significantly modify the relationship between markers of reservoir size or activity in the linear mixed effects models (LME) (**Supplementary Table 2**). However, the LME indicated that female sex was associated with higher CXCL10 values over time on ART (*t*-statistic (*for male*) =-2.6276; p=0.0115). As we only included sex in these models as a covariable to adjust for potential correlations between reservoir size dynamics and immune activation markers we should refrain from overinterpretation of this result. Taken together, these data suggest that while there were notable differences in initial CD4^+^ T cell counts at baseline, these differences did not persist or impact the broader molecular and immunological dynamics measured within our cohort.

## 4 Discussion

HIV-1 remains a significant global health challenge. While antiretroviral therapy effectively controls viral replication, achieving a cure remains an essential but unmet goal. It is crucial to deepen our understanding of the size, characteristics, and impact of the viral reservoir, particularly among women and men infected with HIV-1 subtype C, the most prevalent viral subtype worldwide. This understanding will advance equity and equality in HIV-1 research and healthcare.

Our data suggest that defective proviruses may contribute to HIV-1 transcriptional activity, immune activation, and the regulation of CD4**^+^** T cell counts. We found a significant baseline correlation between the number of defective proviruses and both plasma VL and cell-associated msRNA transcript levels. This aligns with findings indicating that some defective proviruses can still generate viral transcripts and proteins, and that up to 50% of plasma-derived virions are genetically defective (33, 34). Continuous viral replication and the generation of viral products, including defective ones, are thought to contribute to persistent inflammation, activation, and immune dysfunction. We also show a significant impact of the defective proviruses on the immune system, as indicated by correlations with the cytokines CXCL10 and TNF-α. TNF-α, a crucial regulator of immune responses and inflammation, plays a role in inducing CXCL10 expression (35, 36). CXCL10, in turn, is involved in immune responses, inflammation, and plays a vital role in antiviral defense mechanisms (35). Consistent with high levels of immune activation before treatment, CD4^+^ T cell depletion occurs. At baseline, we observed an inverse relationship between the size of defective proviral DNA and CD4^+^ T cell counts, suggesting that the defective proviruses contribute to CD4^+^ T cell depletion.

The intricate relationship between immune activation and viral production is bidirectional and complex. On one hand, immune activation can drive cell activation and viral production (37). Conversely, the production of viral proteins and particles can stimulate the immune system (37). This creates a positive feedback loop wherein immune activation and viral production mutually reinforce each other.

In the context of achieving a cure, our findings underscore the importance of eradicating not only the intact viral reservoir but also the defective proviruses to stabilize immune function. While eliminating intact and replication-competent viruses is crucial for preventing new rounds of infection, the more abundant defective proviruses have the potential to activate the immune system and to contribute to HIV-related co-morbidities (38). These insights enhance our understanding of the defective proviruses, emphasizing that this fraction is far from a dormant repository of inactive viral remnants.

Another significant baseline observation within our cohort is the lower CD4+ T cell count in men compared to women. This discrepancy may be linked to behavioral differences, as previous research has demonstrated that men tend to seek medical care later than women, leading to more advanced HIV-1 infection and consequently lower CD4+ T cell counts (39, 40). Beyond behavioral differences, several studies have reported biological sex differences in CD4+ T cell counts. These differences have been observed across European, Asian, and African populations, with age-matched females showing higher CD4^+^ T cell counts compared to males (41–43). Moreover, various studies have identified biological distinctions in the viral reservoir between men and women. Estrogen and β-Estradiol have been recognized as repressors of proviral reactivation, leading to the hypothesis that women may have a lower frequency of inducible virus (17). Moreover, a recent study suggested a link between the female immune response and the proviral reservoir, finding a greater enrichment of intact proviruses in transcriptionally silent chromosomal locations in females on long-term ART. This suggests that the female immune system may exert a more effective immune-mediated selection pressure against proviruses in accessible euchromatin (44). However, a study conducted by Falcinelli et al., which examined CD4^+^ T cell HIV-1 reservoirs in both men and women under ART suppression, did not find any disparities between the biological sexes (18). Similarly, another study investigating differences in the frequency of total and integrated DNA of CD4^+^ T cells found no apparent variations (19). In line with the aforementioned studies, our own observations indicate that, apart from the CD4^+^ T cell difference at baseline, no further distinctions emerged regarding our VL, plasma immune levels, proviral DNA, and cell-associated msRNA between men and women. Overall, there remains considerable ambiguity and contradictory data in the field of biological sex differences, underscoring the need for further research.

Over two years of treatment, we showed that suppressive antiretroviral therapy resulted in a significantly more pronounced decrease in the intact proviral reservoir compared to defective proviruses. During the first year of therapy, both the number of intact and defective proviruses decreased. In the second year, the number of defective proviruses remained relatively stable, whereas a continuous decline in the intact proviral reservoir was observed.

Additionally, there was a significant increase in CD4^+^ T cell numbers, a decrease in cell-associated HIV-1 msRNA, and a reduction in immune activation markers. The significant increase in IL-2 levels over time can be attributed to activated CD4^+^ T cells producing IL-2, which in turn supports the proliferation and differentiation of T cells (45). Over time, during suppressive antiretroviral therapy, we no longer observed a correlation between defective proviruses and immune activation. We cannot exclude the possibility that viral production still impacts immune activation and vice versa, but this effect might not be measurable due to the lower levels of HIV-1 DNA and immune markers after two years of ART.

Our observations on the decrease over time of intact and defective proviruses in individuals with HIV-subtype C align with findings from other studies involving PWH subtype B. A study conducted by Peluso et al., followed 81 individuals with HIV-1 subtype B for a median duration of 7.3 years and also observed a pronounced decrease of the intact proviral reservoir compared to the defective fraction (46). Moreover, a study performed on acutely treated HIV-1 subtype B infections observed a steep decay for both intact and defective proviruses in the first 5 weeks, followed by a slower decay (47). Furthermore, after 2 decades of treatment, Gandhi et al., reported a selective decay of intact proviruses with little to no decay for the defective proviruses. Additionally, recent studies demonstrated a biphasic decay pattern of the intact reservoir during the first months of ART compared to the decay after 3-4 months of ART. In the initial stage, a half-life of 12.9 days was observed, in contrast to the half-life of 19 months observed after 3-4 months of ART (25). The greater decay of the intact reservoir may be attributed to the cytotoxic effects of virion production by this reservoir (48). Additionally, cells containing intact proviruses are likely to produce proteins and gene products, which could lead to enhanced clearance by the host immune response (25, 46). As indicated above, recent studies suggest that defective proviruses may also generate viral transcripts and proteins which may act as a decoy by diverting immune responses towards defective viruses, potentially resulting in reduced recognition and clearance of cells harboring replication-competent HIV-1 (49). However, the production of defective proteins and viral particles also contributes to chronic immune activation and T cell exhaustion (24, 33).

Our study had certain limitations. Polymorphisms that affect the binding sites of the IPDA primers or probes may result in an underestimation of intact proviruses. However, it is noteworthy that all molecular assays are susceptible to polymorphisms. Our primers and probes were designed to bind to highly conserved regions of HIV-1 subtype B and C, and our annealing temperature makes the assay tolerant to minor polymorphisms without increasing the likelihood of detecting false positives (27). Additionally, due to sample availability, we only measured HIV-1 msRNA, given its association with viral production. Including HIV-1 unspliced RNA, which is more abundantly present than HIV-1 msRNA, could provide extra information about the transcriptional status of the virus (28). Future research would benefit from large blood draws or, preferably, leukapheresis samples to provide more insights into HIV-1 sequences, clonality, and integration sites. This approach would facilitate an even more comprehensive characterization of viral reservoirs, thereby accelerating HIV-1 cure research.

## 5 Conclusion

This study highlights the significant relationship at baseline between the defective proviruses and the activity of the reservoir as measured in both plasma and cell-associated RNA. It also provides evidence linking the defective proviruses to immune response indicators such as CD4^+^ T cell count, CXCL10, and TNF-α. Additionally, the observation that the defective proviruses exhibit a less pronounced decrease during ART underscores the importance of considering defective proviruses alongside the intact reservoir when developing therapeutic strategies to improve clinical outcomes for PWH.

## Data Availability

The raw data supporting the conclusions of this article will be made available by the authors, without undue reservation.
