# Supplementary material for: Defective proviruses significantly impact viral transcription and immune activation in men and women with HIV-1 subtype C in rural South Africa
Source: Front Immunol. 2024 Nov 26;15:1484358. doi: 10.3389/fimmu.2024.1484358 (PMC11628515; doi:10.3389/fimmu.2024.1484358)
Supplement: Supplementary file 1 [file DataSheet1.pdf]

Supplementary Material

1 Supplementary Data

Supplementary table 1: overview of the primers and probes.

**Primer**	**Sequence**	**Position HXB2**
psi forward	TCTCGACGCAGGACTCG	684-700
psi probe	56-FAM/CTCTCTCCT/ZEN/TCTAGCCTC/3IABkFQ	772-789
psi reverse	TATTGACGCTCTCGCACC	793-810
env forward	AGTGGTGGAGAGAGAAAAAAGAGC	7736-7759
env probe	/5HEX/CCTTGGGTT/ZEN/CTTGGGAGC/3IABkFQ/	7781-7798
env hypermutation probe	/5IABkFQ/CCTTAGGTTCTTAGGAGC/3IABkFQ/	7781-7798
env reverse		7851-7832
RPP30 forward 1	GATTTGGACCTGCGAGCG	n/a
RPP30 reverse 1	GCGGCTGTCTCCACAAGT	n/a
RPP30 probe 1	5HEX/CTGACCTGA/ZEN/AGGCTCT/3IABkFQ	n/a
RPP30 forward 2	CCATTTGCTGCTCCTTGGG	n/a
RPP30 reverse 2	CATGCAAAGGAGGAAGCCG	n/a
RPP30 probe 2	/56-FAM/AAGGAGCAA/ZEN/GGTTCTATTGTAG/3IABkFQ/	n/a
msRNA forward	GCAGTRAGGATCATCAA RAT CYTRTATCAAAGC	6012-6044
msRNA probe	56-FAM/CTTCTTCGA/ZEN/TTCYTCCGRGCCTGTC/3IAKbFQ	8406-8430
msRNA reverse	GATCTGYCTYTGTCTTGCTCTCCACCT	8432-8458

Supplementary table 2: Presents the outcomes of the linear mixed effects analysis as performed in R. Abbreviations: viral load (VL), cell-associated multiple spliced RNA(msRNA), Intact Proviral DNA Assay (IPDA), Interleukin (IL), Tumor Necrosis Factor alpha (TNF-α), cluster of differentiation (CD), chemokines C-C motif chemokine ligand 2 (CCL2), C-X-C motif chemokine 10 (CXCL10), c-reactive protein (CRP).

outcomes of the linear mixed effects analysis

		covariables					
		**changes over time (weeks)**	**sex**	**baseline VL (copies/ml)**	**baseline msRNA (copies/µg RNA)**	**baseline CD4$^+$ T cells (cells/mm^3)**	**IPDA total (copies/10^6 cells)**
**VL (copies/ml)**	**std Error**	0.0028	0.2345		0.1275	0.0005	0.2240
	**t-value**	-17.5686	-0.8647		0.0556	-1.1831	2.3627
	**p-value**	**0.0000**	0.3912		0.9559	0.2423	**0.0220**
**msRNA (copies/µg RNA)**	**std Error**	0.0027	0.2162	0.1616		0.0005	0.2308
	**t-value**	-5.0294	0.0993	0.6231		-1.2991	0.7758
	**p-value**	**0.0000**	0.9213	0.5360		0.1998	0.4415
**CD4$^+$T cells (cells/mm3)**	**std Error**	0.2965	56.8023	41.4237	31.2435		67.3983
	**t-value**	7.1673	-1.5961	-3.4093	-0.8500		-0.0800
	**p-value**	**0.0000**	0.1164	**0.0013**	0.3991		0.9366
**IPDA total (copies/106 cells)**	**std Error**	0.0008	0.1080	0.0705	0.0524	0.0003	
	**t-value**	-8.2380	-0.4747	3.7931	2.0223	-0.7826	
	**p-value**	**0.0000**	0.6369	**0.0004**	**0.0481**	0.4373	
**IPDA intact (copies/106 cells)**	**std Error**	0.0016	0.2468	0.1618	0.1197	0.0006	
	**t-value**	-5.2335	-0.2226	1.3664	1.0136	-0.1869	
	**p-value**	**0.0000**	0.8247	0.1775	0.3153	0.8524	
**IPDA defective (copies/106 cells)**	**std Error**	0.0011	0.1214	0.0777	0.0589	0.0003	
	**t-value**	-5.0856	0.2993	3.0167	1.9780	-0.6170	
	**p-value**	**0.0000**	0.7658	**0.0039**	0.0530	0.5398	
**IPDA psi (copies/106 cells)**	**std Error**	0.0011	0.1574	0.1027	0.0763	0.0004	
	**t-value**	-4.4800	0.5775	1.9720	1.0917	-0.5864	
	**p-value**	**0.0000**	0.5660	0.0537	0.2798	0.5600	
**IPDA env (copies/106 cells)**	**std Error**	0.0016	0.2172	0.1411	0.1053	0.0005	
	**t-value**	-2.9187	0.0052	0.9079	2.0087	-0.7555	
	**p-value**	**0.0051**	0.9958	0.3680	**0.0496**	0.4532	
**ratio IPDA intact (%)**	**std Error**	0.0446	6.0716	3.9526	2.9443	0.0141	
	**t-value**	-2.7825	-0.8411	0.8327	-0.47301	0.3992	
	**p-value**	**0.0075**	0.4040	0.4087	0.6381	0.6913	
**IL-2 pg/ml**	**std Error**	0.0010	0.0975	0.0783	0.0527	0.0002	0.1106
	**t-value**	3.5622	0.5672	-0.0490	0.9869	-0.5493	-0.4748
	**p-value**	**0.0006**	0.5732	0.9611	0.3286	0.5853	0.6371
**IL-6 pg/ml**	**std Error**	0.0005	0.1034	0.0850	0.0560	0.0002	0.1202
	**t-value**	-5.1316	-0.5169	-0.3675	-0.5837	0.1091	0.5238
	**p-value**	**0.0000**	0.6076	0.7149	0.5621	0.9135	0.6028
**IL-7 pg/ml**	**std Error**	0.0010	0.1101	0.0888	0.0595	0.0003	0.1253
	**t-value**	-10.3949	0.8560	3.2003	-1.1323	-1.3780	-2.9003
	**p-value**	**0.0000**	0.3962	**0.0024**	0.2631	0.1746	**0.0056**
**IL-12 pg/ml**	**std Error**	0.0001	0.0099	0.0079	0.0053	0.0000	0.0112
	**t-value**	-0.0626	-1.1746	0.0032	0.8317	-0.0558	0.1689
	**p-value**	**0.9503**	0.2460	0.9975	0.4097	0.9557	0.8666
**TNF-a pg/ml**	**std Error**	0.0006	0.0684	0.0554	0.0371	0.0002	0.0782
	**t-value**	-10.3333	0.0418	1.3314	1.5632	-1.6678	-1.4851
	**p-value**	**0.0000**	0.9668	0.1893	0.1246	0.1019	0.1441
**sCD14 pg/ml**	**std Error**	0.0005	0.0432	0.0345	0.0233	0.0001	0.0489
	**t-value**	-4.2996	-1.3790	0.4493	0.4029	-1.9793	-2.5651
	**p-value**	**0.0001**	0.1743	0.6552	0.6888	0.0535	**0.0135**
**sCD163 pg/ml**	**std Error**	0.0003	0.0842	0.0693	0.0456	0.0002	0.0981
	**t-value**	-6.0801	-1.5409	-0.4508	1.1408	-0.1494	0.0066
	**p-value**	**0.0000**	0.1299	0.6541	0.2596	0.8818	0.9947
**CCL2 pg/ml**	**std Error**	0.0003	0.0488	0.0397	0.0264	0.0001	0.0560
	**t-value**	-6.1571	0.0128	0.6659	2.2590	-1.4756	-2.4623
	**p-value**	**0.0000**	0.9898	0.5087	**0.0285**	0.1466	**0.0174**
**CXCL10 pg/ml**	**std Error**	0.0007	0.0662	0.0531	0.0358	0.0002	0.0750
	**t-value**	-5.8999	-2.6276	0.3938	1.8123	-1.1038	-0.8651
	**p-value**	**0.0000**	**0.0115**	0.6955	0.0762	0.2752	0.3913
**CRP pg/ml**	**std Error**	0.0006	0.0717	0.0579	0.0388	0.0002	0.0817
	**t-value**	-2.1475	-0.6693	0.5354	-0.3606	-1.0561	-2.5813
	**p-value**	**0.0350**	0.5065	0.5949	0.7200	0.2962	**0.0130**

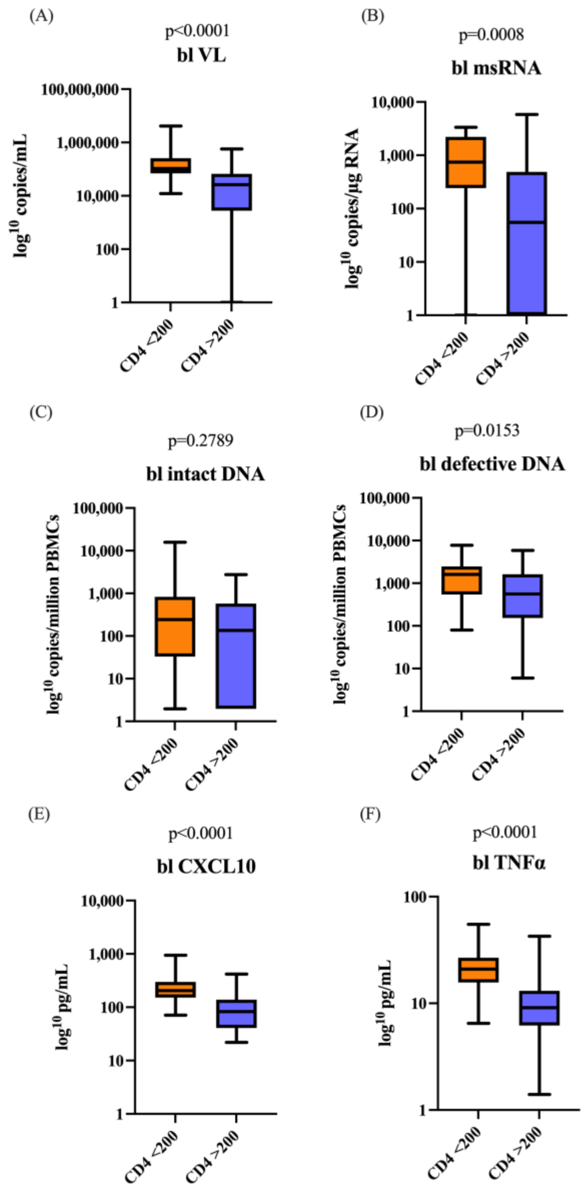

Supplementary figure 1**:** Presents the impact of <200 and >200 CD4+ T cells/mm^3 before the start of ART on **(A)** VL, **(B)** msRNA, **(C)** intact proviral DNA and **(D)** defective proviral DNA. The boxplots present (from top to bottom) the maximum, third quartile, median, first quartile, and minimum values. The dataset was log transformed and the analysis was performed with a Mann-Whitney U test in GraphPad.

Abbreviations: viral load (VL), cell-associated multiple spliced RNA(msRNA), Intact Proviral DNA Assay (IPDA), Tumor Necrosis Factor alpha (TNF-α), C-X-C motif chemokine 10 (CXCL10).
